# Characterization of Non-Obstructive Azoospermia in Men Using Gut Microbial Profiling

**DOI:** 10.3390/jcm12020701

**Published:** 2023-01-16

**Authors:** Yalei Cao, Haojie Wang, Zirun Jin, Jing Hang, Hui Jiang, Huajun Wu, Zhe Zhang

**Affiliations:** 1 Department of Urology, Peking University Third Hospital, Beijing 100191, China; 2 Institute of Genetics and Developmental Biology, Chinese Academy of Sciences, Beijing 100101, China; 3 Center for Reproductive Medicine, Department of Obstetrics and Gynecology, Peking University Third Hospital, Beijing 100191, China; 4 Key Laboratory of Assisted Reproduction, Ministry of Education, Beijing 100191, China; 5 Beijing Key Laboratory of Reproductive Endocrinology and Assisted Reproduction, Beijing 100191, China; 6 National Clinical Research Center for Obstetrics and Gynecology, Beijing 100191, China; 7 Department of Urology, Peking University First Hospital, Institute of Urology, Beijing 100034, China; 8 Center for Precision Medicine Multi-Omics Research, School of Basic Medical Sciences, Peking University Health Science Center, Peking University Cancer Hospital and Institute, Beijing 100191, China

**Keywords:** non-obstructive azoospermia, gut microbiota, shotgun sequencing, male infertility

## Abstract

(1) Background: Non-obstructive azoospermia (NOA) is a complex multifactorial disease and the causes of most NOA cases remain unknown. (2) Methods: Here, we performed comprehensive clinical analyses and gut microbial profiling using shotgun metagenomic sequencing in patients with NOA and control individuals. (3) Results: The gut microbial alpha and beta diversity significantly differed between patients with NOA and controls. Several microbial strains, including *Bacteroides vulgatus* and *Streptococcus thermophilus*, were significantly more abundant in the NOA group, whereas *Bacteroides thetaiotaomicron* and *Parabacteroides* sp. *CT06* were enriched in the control group. Moreover, functional pathway analysis suggested that the altered microbiota in NOA suppressed the carbohydrate metabolism pathway, while amino acid metabolism and methane metabolism pathways were enhanced. We observed that the differential microbial species, such as *Acinetobacter johnsonii*, had a strong correlation with clinical parameters, including age, body mass index, testosterone, and follicle-stimulating hormone. Communication and interplay among microbial genera were significantly increased in NOA than in the control group. (4) Conclusions: Altered microbial composition and functional pathways in the NOA group were revealed, which highlight the utility of gut microbiota in understanding microbiota-related pathogenesis of NOA and might be helpful to the clinical management of NOA.

## 1. Introduction

Infertility is a global problem affecting approximately 12% of couples in the reproductive-age, with male infertility factors accounting for nearly 50% of them [1]. Non-obstructive azoospermia (NOA), detected in 10–15% of infertile men, is one of the most common causes of male infertility [2,3]. Patients with NOA have primary or secondary testicular failure with various underlying etiologies, including genetic disorders, hormonal anomalies, chemotherapy or radiation treatment, infection, and inflammation. Congenital factors, such as Klinefelter syndrome and microdeletions in the AZF region of the Y chromosome, can also cause NOA, but the causes of most NOA cases remain unknown; these cases are collectively termed idiopathic NOA (iNOA). Although current novel clinical testing including whole exon sequencing and whole genome sequencing could partly uncover the causes of iNOA in some cases, the pathological mechanism underlying iNOA remains unclear [4]. Hence, comprehensive etiological analyses are urgently required for effective treatment of patients with NOA.

Gut microbiota, as the second largest genome of the host, is associated with disorders of the testis, liver, brain, immune cells, and certain endocrine gland [5,6,7,8]. To date, several studies have characterized the gut microbiome profile in relation to female fertility, and bacterial dysbiosis has been linked to reproductive diseases [9,10]. The dysbiosis in the gut and genitourinary microbiota is associated with idiopathic male infertility [11]. Moreover, the increased prevalence of *Bacteroides* and *Prevotella* resulted in a decrease in spermatogenesis and sperm motility in a high-fat-diet-fed fecal microbiota transplantation mouse model [12]. These findings indicate that gut microbiota may affect male reproduction at multiple levels. However, systematic and complete research on the alterations in the gut microbiota and its role in NOA is still lacking.

The current study used shotgun metagenomic sequencing and analysis to provide high-resolution taxonomic and functional profiles involved in the development of iNOA. We also performed a correlation analysis between clinical parameters and gut microbiota, as well as the functional differences in the corresponding microbial communities. This work will shed new light on the causes of male infertility and the clinical management of iNOA.

## 2. Materials and Methods

### 2.1. Collection of Human Plasma and Fecal Samples

Plasma and fecal samples were obtained from donors, including healthy males and patients with NOA, undergoing routine semen analyses in our clinical laboratory. Sperm quality was assessed using a computer-assisted semen analysis (CASA, Suijia Company, Beijing, China) following the guidelines of the 5th WHO laboratory manual for the examination and processing of human semen. The criteria for normal sperm of healthy males were sperm concentration >15 × 10^6^/mL, progressive motility >32% and total motility >40%. The following criterion was considered to diagnose NOA: absence of sperm in the ejaculate, confirmed by centrifuging the semen specimen three times. The patients with NOA received testicular sperm extraction and those with bilateral distal or proximal obstruction of the ejaculatory ducts (obstructive azoospermia) were excluded. In our study, the phrasing “patients with NOA” refers to idiopathic NOA; we excluded patients with NOA where the condition was caused by Klinefelter syndrome, microdeletions of the Y chromosome, cryptorchidism, orchitis, chemoradiotherapy, and urinary tract infection. The exclusion criteria were as follows: (1) abnormal liver, kidney, or heart function; (2) presence of gastrointestinal disease, active infections, thyroid disorders, hypertension, or diabetes mellitus; (3) administration of oral probiotics or antibiotic within at least 3 months before the study.

This study was approved by the institutional review board of Peking University Third Hospital (approval number 2017SZ-035). All participants provided informed consent for use of their clinical data and fecal samples.

### 2.2. Laboratory Measurements

Blood samples were collected when patients with NOA were admitted to the hospital. Serum testosterone and FSH levels were measured using an automated chemiluminescent immunoassay (Siemens IMMULITE 2000 immunoassay system; Siemens Healthcare Diagnostics, Shanghai, China).

### 2.3. Sample Preparation and DNA Extraction

Fresh fecal samples collected from patients with NOA and healthy donors were immediately frozen using liquid nitrogen and stored at −80 °C until further analysis. Genomic DNA from fecal samples was extracted using a QIAGEN kit and analyzed using 1% agarose gel electrophoresis. The quality of the DNA samples was further assessed using a Qubit^®^ 2.0 fluorometer (Life Technologies, Carlsbad, CA, USA). Samples with an OD value of 1.8–2.0 were used for library construction, and 1 μg DNA per sample was used as input material for DNA sample preparation.

### 2.4. Shotgun Metagenomic Sequencing

Sequencing libraries were generated using NEBNext^®^ UltraTM DNA Library Prep Kit for Illumina (NEB, Ipswich, MA, USA) following the manufacturer’s recommendations, and index codes were added to attribute sequences of each sample. Briefly, DNA samples were fragmented to a size of 350 bp via sonication, and the DNA fragments were then end-polished, A-tailed, and ligated with the full-length adaptor for Illumina sequencing and further PCR-amplified. The PCR products were purified (AMPure XP system, San Diego, CA, USA), and libraries were analyzed for size distribution using an Agilent 2100 Bioanalyzer and quantified using real-time PCR. After clustering of the index-coded samples cluster generation on a cBot Cluster Generation System according to the manufacturer’s instructions, the library preparations were sequenced on an Illumina NovaSeq platform, and paired-end reads were generated, with at least 10 Gb reads per sample being generated. The metagenomic sequencing raw reads generated during this study are freely available at SRA (https://www.ncbi.nlm.nih.gov/sra/, accessed on 5 December 2022) under the accession number PRJNA905731.

### 2.5. Pretreatment of the Sequencing Results and Metagenome Assembly

To acquire clean data for subsequent analyses, we used Readfq (Broad Institute of Harvard and MIT, Cambridge, MA, USA, v8, https://github.com/cjfields/readfq, accessed on 1 November 2022) to filter. Host sequences were discarded by mapping the sequences against the reference genome (hg19) using BowTie2.2.4 (SourceForge, San Diego, CA, USA, http://bowtie-bio.sourceforge.net/bowtie2/index.shtml, accessed on 1 November 2022). To construct the gene catalog, we pooled and subjected the remaining set of clean reads to metagenomes using the SOAPdenovo software (SourceForge, San Diego, CA, USA, v2.04, http://soap.genomics.org.cn/soapdenovo.html, accessed on 1 November 2022). We then interrupted the assembled Scaftigs from N connection and removed all Scaftigs without N. Using Bowtie2.2.4 (SourceForge, San Diego, CA, USA), the clean data of all samples were compared to the corresponding scaffold to acquire the PE reads that were not used.

### 2.6. Gene Prediction and Abundance Analyses

Scaftigs (>500 bp) were assembled and used for prediction of the open-reading frame, using MetaGeneMark (Georgia Institute of Technology, Atlanta, GA, USA, v2.10, http://topaz.gatech.edu/GeneMark/, accessed on 3 November 2022), and the length information shorter than 100 nt was filtered. CD-HIT (v4.5.8, http://www.bioinformatics.org/cd-hit, accessed on 3 November 2022) was used for constructing the unique initial gene catalog and conducting the open-reading frame prediction.

### 2.7. Taxonomy Prediction

DIAMOND (v0.9.9, https://github.com/bbuchfink/diamond/, accessed on 4 November 2022) was used to blast unigenes to bacterial, fungal, archaeal, and viral sequences extracted from the NR database (v2018-01-02, https://www.ncbi.nlm.nih.gov/, accessed on 4 November 2022). The LCA algorithm was applied to the system classification of MEGAN to verify the species annotation information of sequences.

### 2.8. Common Functional Database Annotations

We used DIAMOND (v0.9.9) to blast unigenes to functional databases, including KEGG (v2018-01-01, http://www.kegg.jp/kegg/, accessed on 4 November 2022), eggNOG (v4.5, http://eggnogdb.embl.de/#/app/home, accessed on 4 November 2022), and CAZy (v201801, http://www.cazy.org/, accessed on 4 November 2022). For each sequence’s blast result, the one with the best blast hit was used for subsequent analysis.

### 2.9. Co-Occurrence Network Analysis

To predict interactions between different gut bacterial species of the NOA and control groups, co-occurrence patterns of core bacterial genera were evaluated using pairwise Spearman’s rank correlations (rs) based on relative bacterial abundance. The Spearman’s rank correlations were analyzed using the Hmisc package in R. A significant rank correlation between two genera (rs > 0.25 or rs < −0.25, false discovery rate-adjusted *p* < 0.001) was considered to be a co-occurrence event. The network was visualized using the igraph package in R (http://igraph.org, accessed on 5 November 2022). The most densely connected node in each network was defined as the “hub,” which was an indicator of keystone species, integral for maintaining connectivity within the network.

### 2.10. Statistical Analyses

To analyze the statistical significance of different taxonomic levels (phylum, genus, and species), genes, and KEGG orthologs, we used the nonparametric Wilcoxon test (“wilcox.” test in R). The Benjamini–Hochberg method correction was used in multiple comparisons. Enriched features with *p* < 0.05 were identified, and enriched groups were identified based on higher rank sum values. The linear discriminant analysis (LDA) effect size (LEfSe) method was used to identify the organisms best suited to explain differences between groups. Different organisms with an LDA score cutoff of 3.0 were identified. For continuous variables, the differences between groups were analyzed using independent-sample *t*-tests, and the mean ± standard deviation in each group was analyzed. For categorical variables, the Fisher’s exact test was used at a two-sided significance level of 0.05.

## 3. Results

### 3.1. Clinical Parameters of NOA and Control Groups

**Figure 1:**
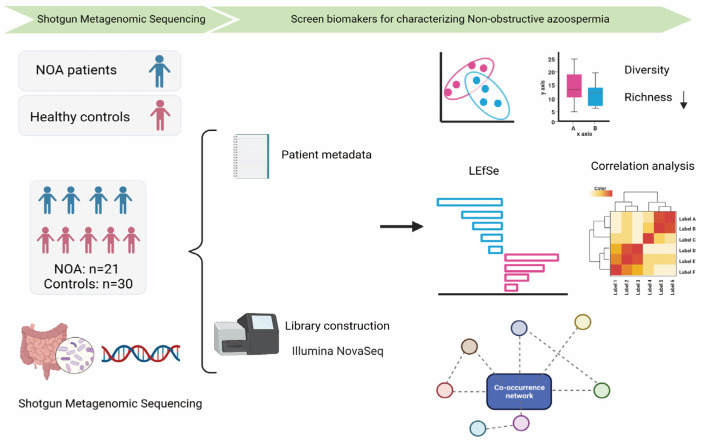
Scheme of the study. We collected comprehensive clinical data and performed gut microbial profiling through shotgun metagenomic sequencing in 21 patients with iNOA and 30 control individuals. We intended to find biomarkers for characterizing NOA. iNOA: idiopathic non-obstructive azoospermia.

We recruited 21 patients with iNOA and 30 healthy subjects (Figure 1). Baseline demographics are shown in Table 1. Although we have analyzed sperm quality of the healthy donors by CASA, the data of morphology of sperm is lacking. There was no significant difference in the age between the two groups (29.43 ± 6.20 vs. 32.14 ± 3.81, control vs. NOA). However, the body mass index (BMI; *p* = 0.05) and levels of testosterone (*p* = 0.002) and follicle-stimulating hormone (FSH; *p* < 0.001) differed between the two groups. Patients with NOA had higher BMI, higher levels of FSH, and lower levels of testosterone compared with those of the control group.

### 3.2. Microbial Composition and Diversity of Gut Microbiota

**Figure 2:**
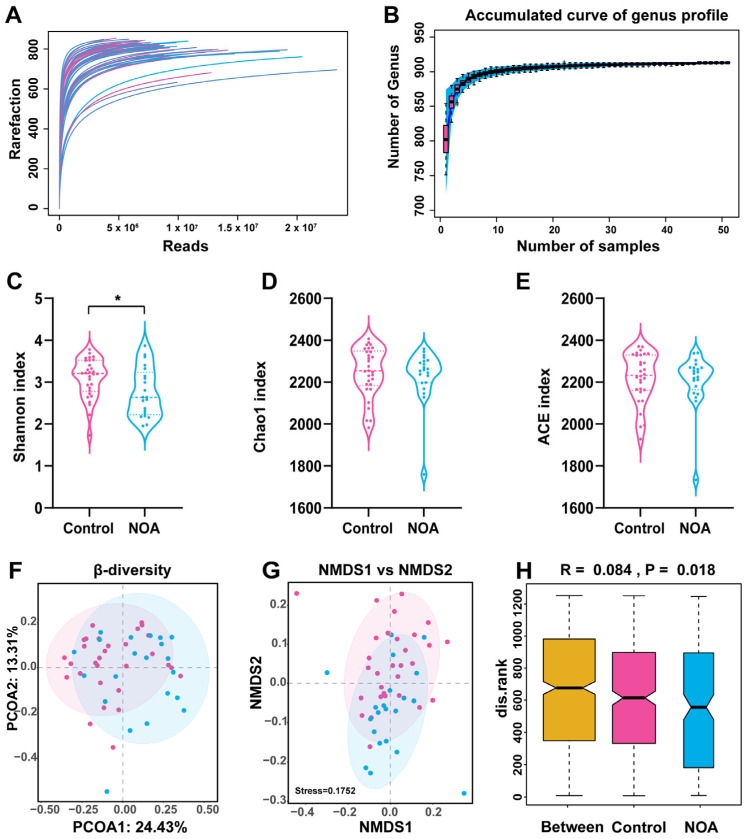
Overview of the gut microbiome in the two experimental groups. Gut microbial diversity was analyzed on the basis of shotgun metagenomic sequencing. Rarefaction curves of reads (**A**) and accumulation curve of genus profile (**B**) are presented. Shannon index (α-diversity) (**C**), Chao1 index (**D**), and ACE index (**E**) were used to describe α diversity of gut microbiota in the two groups. Bacterial command compositional similarity was evaluated by β diversity. Plots of principal coordinate analysis (PCoA) with Bray–Curtis distance (**F**) and non-metric multidimensional scaling (NMDS) (β-diversity) (**G**) at species levels are shown. Analysis of similarities (Anosim) (**H**) was used to detect differences between the two groups. * indicates significance (*p* < 0.05) when control group is compared with NOA group. Pink represents control group and blue represents NOA group.

To comprehensively characterize the gut microbial community structure in patients with NOA, we carried out high throughput shotgun metagenomic sequencing. The accumulation and rarefaction curves indicated adequate sample size and depth of analysis (Figure 2A,B). With increasing sample size, the number of detected genera reached close to 950 and the interquartile range became smaller. The curve began to plateau when the sample size reached 51, indicating that the sample size was adequate.

Next, we analyzed the α-diversity and β-diversity to determine the differences in the microbial communities between the two groups. The Shannon index (α-diversity) of the microbiota in the NOA group was significantly lower than that in the control group (Figure 2C). However, the Chao1 index (Figure 2D) and ACE index (Figure 2E) showed no difference between the two groups. Furthermore, principal coordinate analysis and non-metric multidimensional scaling analysis demonstrated distinct β-diversity in the composition of the gut microbiota between the two groups (Figure 2F–G). The Permanova/Anosim analysis showed significant differences between the microbiomes of two groups (Figure 2H). Together, these results suggest that α- and β-diversity of gut microbiota differed in the NOA and control groups.

### 3.3. Different Microbial Species between NOA and Control Groups

**Figure 3:**
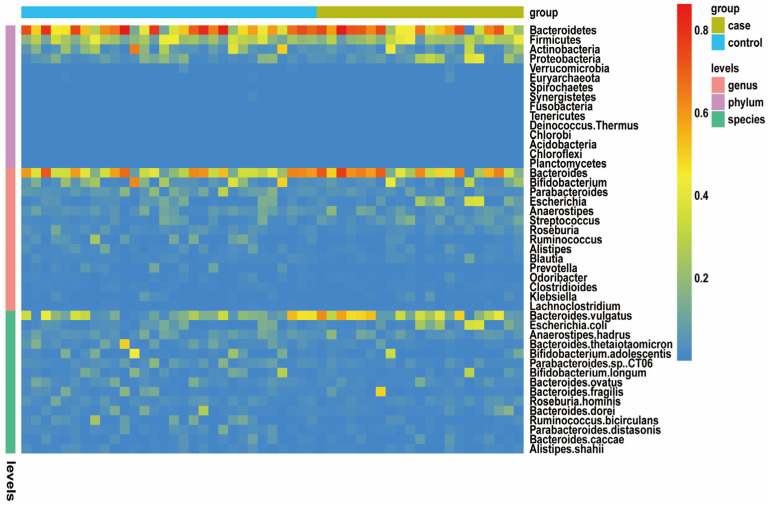
Taxonomic features of the gut microbiota of the healthy control (*n* = 30) and patients with NOA (*n* = 21). An overview of the 15 most abundant phyla, 15 most abundant genera, and 15 most abundant species in the stool samples. The taxa levels are presented by relative abundance. Gut microbiota abundance is represented by color gradient. Red indicates high abundance, and blue indicates low abundance.

To detect the taxonomic composition of gut microbiota of the two groups, we compared the variety and relative abundance of individual taxon. A broad overview of the taxonomic data of all subjects is provided in Figure 3. *Bacteroidetes*, *Firmicutes*, *Actinobacteria*, *Proteobacteria*, and *Verrucomicrobia* were the five dominant phyla in both groups. The *Bacteroides* and *Bifidobacterium* were the relatively prevalent genera among microbiota of all subjects. We identified the top 3 prevalent bacterial species in the two groups, which included *Bacteroides vulgatus*, *Escherichia coli*, and *Anaerostipes hadrus*. Hence, these results confirmed that dysbiosis occurred in gut microbiota of patients with NOA.

**Figure 4:**
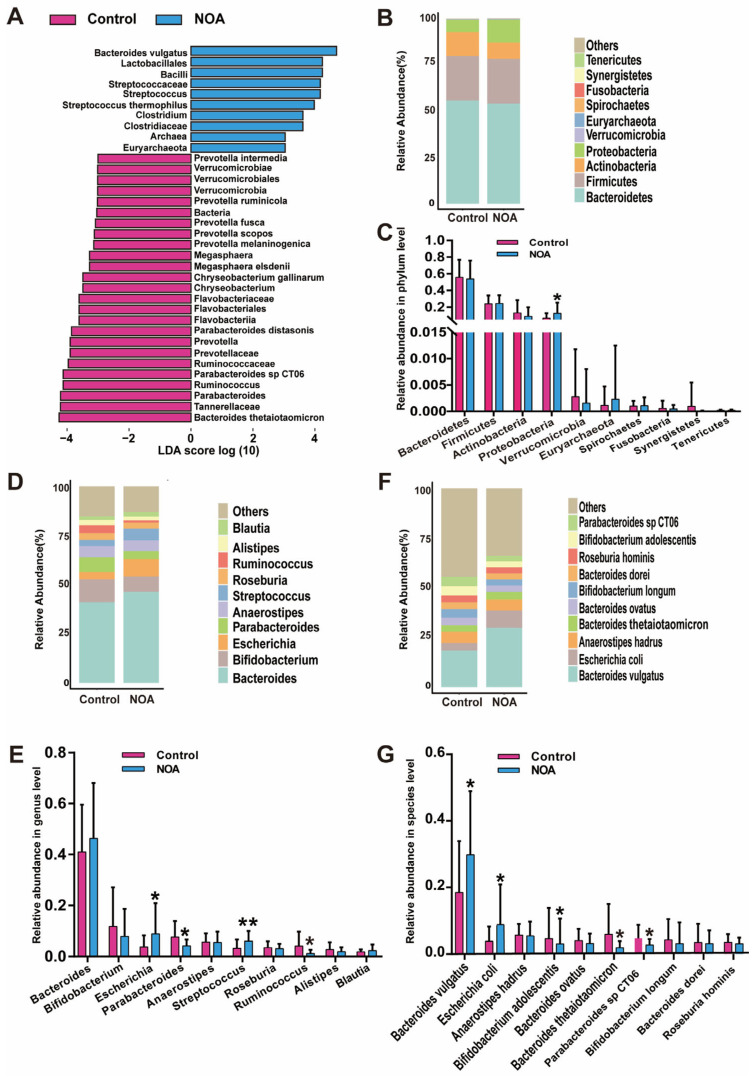
Changes in the taxonomic composition of microbial communities. (**A**) Linear discrimination analysis (LDA) effect size (LEfSe) identified the statistical differences in the levels of biomarkers between the control and NOA groups. The LDA scores (log 10) > 3 are listed. The top ten bacteria, with maximum abundance of fecal bacteria at the phylum (**B**), genus (**D**), and species (**F**) levels. Significant changes in abundance at the phylum (**C**), genus (**E**), and species (**G**) levels; (pink) taxa enriched in the control group, (blue) taxa enriched in the NOA group. Values represent means ± SEM. * indicates significance (*p* < 0.05), ** indicates significance (*p* < 0.01) when control group is compared with NOA group.

To further explore the differences in microbiota of the NOA and control groups at a phylum-to-species level, we used the linear discriminant analysis (LDA) effect size (LEfSe) method of the bacterial contribution degree and statistical testing (Mann–Whitney U test, two-tailed). At the phylum level, *Verrucomicrobia* was enriched in the control group and *Euyarchaeota* was in the NOA group. At the genus level, *Megasphaera*, *Chryseobacterium*, *Prevotella*, *Ruminococcus*, and Parabacteroides were enriched in the control group, whereas *Streptococcus* and *Clostridium* were enriched in the NOA group (Figure 4A). Moreover, several bacterial species, including *B. thetaiotaomicron*, *Parabacteroides* sp. *CT06*, *Parabacteroides distasonis*, and *Chryseobacterium gallinarum*, were enriched in the control group, whereas *B. vulgatus* and *Streptococcus thermophilus* were prevalent specifically in the NOA group (Figure 4A). In addition, our metagenomics data analyzed the gut microbial composition at the phylum, genus, and species levels (Figure 4B–G). The abundance of *Proteobacteria*, *Escherichia*, and *Streptococcus* genera as well as that of the species *B. vulgatus* and *E. coli* were more frequent in the NOA group than in the control group. Conversely, the abundance of genera *Parabacteroides* and *Ruminococcus* and a few species, such as *Bifidobacterium adolescentis*, *B. thetaiotaomicron*, and *Parabacteroides* sp. *CT06*, was significantly decreased in the NOA group compared to that in the control group (Figure 4G). These results revealed the statistically significant difference in microbial phylum, genus, and species found in both groups.

### 3.4. Co-Occurrence Network Analysis of Gut Microbiota in Control and NOA Group

**Figure 5:**
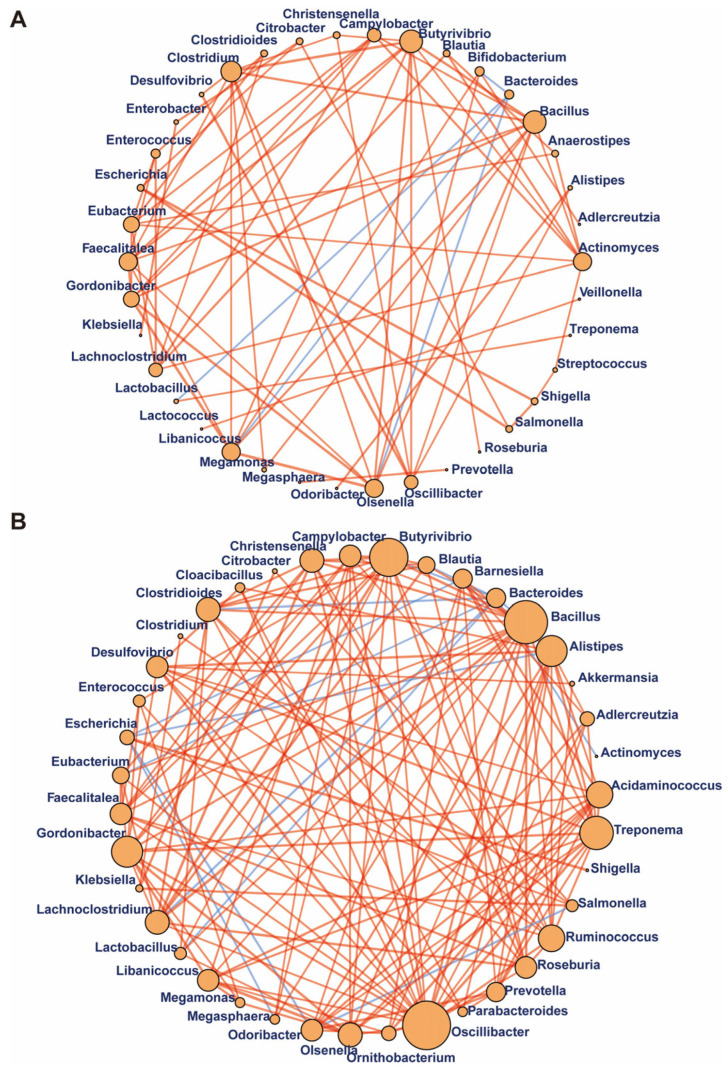
Gut bacterium–bacterium correlation networks in control and NOA groups. Significant positive (orange) and negative (blue) pairwise correlations between different bacteria at the genus level in the control group (**A**) and NOA group (**B**), respectively. Network nodes indicated different core bacterial genera, and edges represented the significant correlation among the nodes. The size of the nodes reflects the degree of connection.

To explore potential reciprocal interactions among bacteria of control and NOA groups, we performed a correlation network analysis through evaluation of pairwise Spearman’s rank correlations of the relative abundances. Most correlations of both groups were positive correlations, suggesting that the ecosystems were primarily characterized by microbial cooperation rather than competition (Figure 5). The correlation network in samples of patients with NOA was stronger than that of the control group because there were more edges, higher mean degree, and transitivity in the NOA group. Moreover, *Bacteroides* was negatively correlated with *Bifidobacterium*, *Lactobacillus*, *Libanicoccus*, and *Olsenella* in control group; while *Butyrivibrio* showed robust correlations with *Bacillus*, *Treponema*, *Clostridioides*, *Gordonibacter*, and *Ruminococcus*. *Oscillibacter* showed substantially more correlations with *Butyrivibrio*, *Bacillus* and other genera in NOA group than in control samples. Therefore, ecological network of the gut microbiome suggested that communication and interplay among genera were significantly altered in NOA.

### 3.5. Functional Annotation of the Bacterial Communities and Its Association with Clinical Parameters

**Figure 6:**
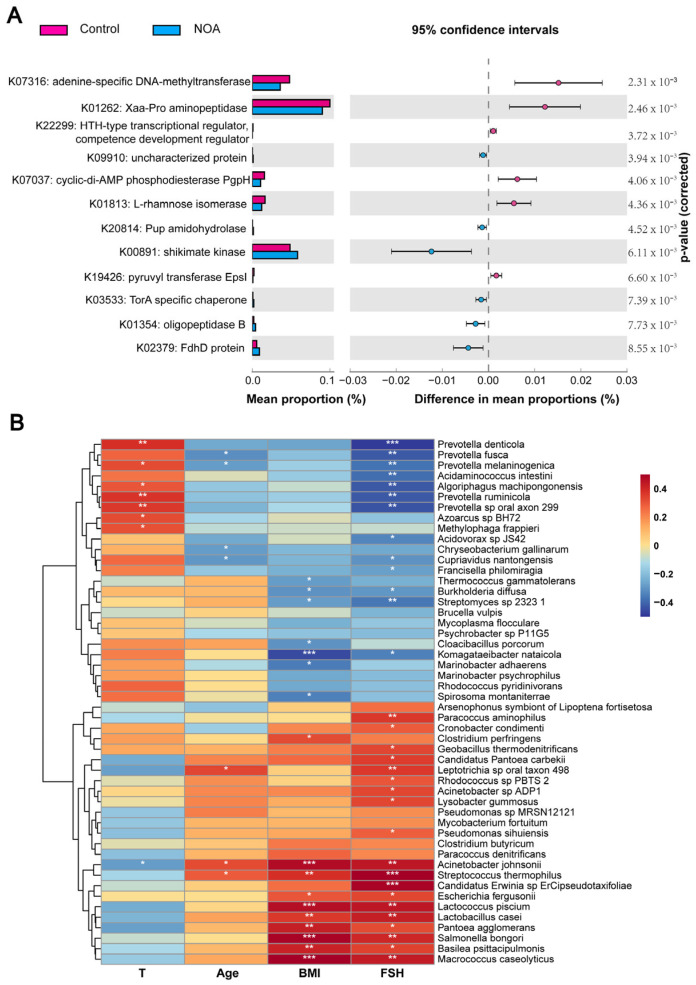
Functional characterization of microbiome and correlation analyses between gut microbiota and clinical parameters. (**A**) Pairwise comparison between control (pink) and NOA (blue) samples demonstrating differences in KEGG orthology (KO) (*p <* 0.01) shown as mean proportion (%) and difference in mean proportions (%) with 95% confidence intervals; *p* values are shown after correcting for negative controls. (**B**) Heat map of the correlation between differential microbiome at the species level and clinical parameters of NOA. Spearman’s rank correlation coefficient is indicated using a color gradient. Red represents positive correlation and blue represents negative correlation. * *p <* 0.05, ** *p <* 0.01, *** *p <* 0.001.

To gain further insights into the underlying regulatory network, we performed functional pathway analysis using shotgun metagenomic data with the HUMAnN2 pipeline [13]. We identified 109 differentially abundant KEGG orthologs (KOs); the top 12 differentially abundant KOs (*p <* 0.01) are listed in Figure 6A, and we found that the most differentially expressed pathway between the two groups was the adenine-specific DNA-methyltransferase pathway. Moreover, the carbohydrate metabolism pathway (such as K01813) was suppressed, while amino acid metabolism and methane metabolism pathways (such as K00891 and K03533) were enriched in the NOA group compared to that in the control group.

The relationship between differential structure of microbiota and clinical parameters was further analyzed. Spearman analysis showed that four species, including *Prevotella fusca* and *Prevotella melaninogenica*, were negatively correlated with age of the subjects while three species, including *Acinetobacter johnsonii* and *S. thermophilus*, had a negative association. In addition, seven species, including *Thermococcus gammatolerans* and *Burkholderia diffusa* were negatively correlated, whereas ten species including *A. johnsonii* and *S. thermophilus* had a positive relationship with BMI (Figure 6B). Furthermore, seven species, including *Prevotella denticola* and *P. melaninogenica*, were positively correlated with testosterone levels, whereas only *A. johnsonii* was negatively correlated with testosterone levels. Negative correlation with FSH levels was detected in 13 species, including *P. denticola* and *P. fusca* (Figure 6B). Together, these results indicate that the differential microbiota in patients with NOA were prominently correlated with clinical parameters.

## 4. Discussion

In the present study, we observed gut microbial dysbiosis in patients with NOA and found a different microbial composition and altered α- and β-diversity in fecal samples of patients with NOA compared with that in samples of their control counterparts. Moreover, we identified many different microbial strains in the NOA and control groups and that the altered gut microbiota had strong correlations with clinical parameters, including age, BMI, and FSH and testosterone levels. Various metabolic pathways, such as glycometabolism, lipid metabolism, and amino acid metabolism, were disrupted in patients with NOA. Our results suggested that the gut microbiome plays an important role in the development of NOA.

Spermatogenesis is a complicated and orchestrated process that involves self-renewal and differentiation of germ cells that may be affected by genetic defects, environmental alterations, and metabolic disorders. Studies have demonstrated an association between spermatogenesis and gut or testicular microbiome [11,12,14]. The transplantation of fecal microbiota from alginate oligosaccharide (AOS)-treated mice to busulfan-treated mice increased the abundance of beneficial intestinal bacteria, such as *Bacteroidetes*, *Bifidobacterium*, *Sphingomonas*, and *Campylobacter*, which facilitated intestinal barrier protection and antioxidant compound production and enhanced antioxidant enzyme production, thus promoting spermatogenesis [15]. Transplantation of *P. distasonis*, implicated in polyamine synthesis, increased polyamine levels and reversed triptolide-induced testicular dysfunction in antibiotic-treated mice [16]. Taken together, it is crucial to know whether patients with spermatogenesis disorders could be affected by altered gut microbiota.

Deep shotgun metagenomics sequencing of fecal samples revealed that the α-diversity and abundance of the gut microbiota were decreased in the NOA group; specifically, the abundance of beneficial gut microbes such as *Ruminococcus bicirculans* and *B. thetaiotaomicron* was decreased in the NOA group compared with that in the control group. *R. bicirculans* can selectively utilize certain hemicelluloses, especially β-glucans and xyloglucan, to degrade plant cell walls and release soluble glucans, which facilitates the absorption of plant nutrients [17]. *B. thetaiotaomicron* can digest several complex carbohydrates and produce short-chain and organic acids, which enhance the mucosal barrier and alleviate inflammation in gastrointestinal disease [18,19]. *Bacteroides vulgatus* was also enriched in the NOA group. Reportedly, *B. vulgatus* is enriched in the gut microbiota of patients with PCOS, and *B. vulgatus* colonization induces metabolic abnormality and ovarian dysfunction in recipient mice [9]. The increased abundance of *Bacteroides* was found to be positively associated with elevated circulating endotoxin levels and contributed to disrupted spermatogenesis and sperm motility in high-fat diet-fed mice [12,20]. Therefore, the ability of *Bacteroides* to transform the metabolism profile in vivo indicates its possible role in inducing spermatogenic disorders. Additionally, two genera of the class *Clostridia* (i.e., *Anaerococcus* and *Peptoniphilus*) have been found to be associated with human sperm motility and morphology [21,22]. The reduction of *Clostridia* in the testicular microbiome is associated with a low sperm retrieval rate in men with iNOA [14]. In the present study, *Clostridium spp.* were enriched in gut microbiota in the NOA group. These data suggest that disrupted gut microbiota is associated with male infertility. Studies found that many fecal bacteria species may serve as a non-invasive biomarker to predict some diseases, such as gestational diabetes mellitus and autism spectrum disorder [23,24]. The application of cell-free nucleotides and proteomics as promising biomarker have been investigated in diagnosis and prognosis of NOA [25,26]. Our finding suggested that *Bacteroides vulgatus* and *Streptococcus thermophilus*, which were significantly more abundant in the NOA group, may be used as potential biomarkers of NOA.

Many patients with iNOA have endocrine disorders apart from disrupted spermatogenesis. In this study, we found higher FSH and lower testosterone levels in the NOA group compared with the control group. Accumulating evidence has shown that gut-derived lipopolysaccharide (LPS) promotes immune response and influences sex hormone secretion. Moreover, bacterial translocation may lead to chronic inflammation, endothelial damage, and disrupted blood–testis barrier (BTB) and thus result in impaired sperm production [27,28]. Microbial-associated molecular patterns, such as LPS, lipoprotein acids, peptidoglycans, and lipoproteins, are absorbed and reach the testis, thus causing testicular damage [29]. Endotoxins inhibit steroid hormone synthesis in Leydig cells and reduce pituitary LH drive and thus result in suppressed testosterone and sperm productions. Gut microbiota and androgen concentrations are correlated; elevated serum testosterone levels and metabolomics changes have been observed when female mice received male gut microbiota [30]. Parabacteroides abundance increases in male mice compared with that in females mice and is positively associated with the testosterone level [30,31]. By contrast, we found a decreased abundance of *Parabacteroides* accompanied with decreased testosterone level in patients with iNOA. Hannah et al. found that the gut microbiota in the cecum can deglucuronidate high levels of glucuronidated dihydrotestosterone and testosterone in the small intestinal content, indicating that probiotics or altered diet may affect gut microbiota composition and modulate intestinal androgen metabolism [32]. Thus, our findings underscore a possible influence of the differentially abundant intestinal microbiota in the onset of NOA.

We further investigated metagenomic functional changes associated with NOA and identified various disrupted metabolic pathways. Features associated with dysregulation of adenine-specific DNA-methyltransferase were found to be substantially different between patients with NOA and controls. In addition, patients with NOA had lower abundances of functional features associated with carbohydrate metabolisms such as K01813 for fructose and mannose metabolism but increased abundances of amino acid metabolism (such as K00891) and methane metabolism (such as K03533) pathways. K00891 is representative of shikimate kinase involved in phenylalanine, tyrosine, and tryptophan iosynthesis. Phenylalanine and tyrosine are associated with hypertension, impaired fasting glucose, and the development of diabetes [33,34]. The microbiome may influence the signaling mechanisms of vitamins A and K, change the biochemical components of the basement membrane in the testis, and induce male infertility [35,36]. AOS—a degradation product of alginate treatment—could increase the abundance of *Lactobacillaceae* and *Desulfovibrionaceae* that are involved in the modification of blood metabolites of AOS, thereby promoting sperm production [37]. Hence, these results provide insights into the probable microbiota pathways attributing to the development of NOA.

Although this pilot study yielded some insightful results, several limitations exist. An important limitation is the small sample size that consequently limited the effect size and statistical power of our results, thereby highlighting the need for further follow-up validation studies on a larger cohort. Although we tried our best to eliminate the interference of infectious diseases, oral probiotics, and antibiotics, studies with more strict criteria including controlled environment, physical activity, lifestyles, and diets are needed for obtaining accurate data. Finally, while we could identify significant microbes related to NOA, their precise mechanistic role in NOA development remains unclear. In-depth studies of each microbe are warranted to confirm the obtained results.

## 5. Conclusions

In conclusion, the present study demonstrates microbiota dysbiosis and microbial changes associated with iNOA, whereby *B. vulgatus* is enriched and *B. thetaiotaomicron* is depleted in fecal samples of patients with iNOA. We also found an association between gut microbiota and clinical parameters. Our results suggest a vital role of the gut microbiota in the development of NOA. Together, our study sheds light on the gut microbiota profile of iNOA and facilitates better understanding of the pathological progress of infertility caused by iNOA.

## Figures and Tables

**Table 1:** Characteristics of the study cohort.

Cohort Characteristics	Control Group (*n* = 30)	NOA Group (*n* = 21)	*p* Value
Age (yr)	29.43 ± 6.20	32.14 ± 3.81	0.060
Body mass index (kg/m^2^)	22.93 ± 2.14	26.33 ± 3.19	0.042 ^a^
Testosterone (nmol/L)	11.59 ± 2.16	8.78 ± 4.06	0.002 ^b^
FSH (IU/L)	12.67 (9.58, 14.98)	26.2 (19.45, 38.10)	<0.001 ^c^
**Semen analysis**			
Semen volume (mL)	2.9 ± 0.98	NA	NA
Sperm concentration (million/mL)	87 (75, 112.75)	NA	NA
% Motile sperm	62.5 (56.75, 66)	NA	NA
Total sperm count (million)	266.25 ± 90.05	NA	NA
Total motile sperm count (million)	165.24 ± 63.65	NA	NA

Data represents mean ± standard deviation or median (interquartile range). BMI = body mass index; FSH = follicle-stimulating hormone; NA = not available. ^a^ *p* < 0.05; ^b^ *p* < 0.01; ^c^ *p* < 0.001.

## Data Availability

The metagenomic sequencing raw reads generated during this study are freely available at SRA (https://www.ncbi.nlm.nih.gov/sra/, accessed on 5 December 2022) under accession number PRJNA905731.
